# Identification of Variable Importance for Predictions of Mortality From COVID-19 Using AI Models for Ontario, Canada

**DOI:** 10.3389/fpubh.2021.675766

**Published:** 2021-06-21

**Authors:** Brett Snider, Edward A. McBean, John Yawney, S. Andrew Gadsden, Bhumi Patel

**Affiliations:** ^1^School of Engineering, University of Guelph, Guelph, ON, Canada; ^2^Adastra Corporation, Toronto, ON, Canada

**Keywords:** artificial intelligence, XGBoost, SHapley, COVID-19, mortality

## Abstract

The Severe Acute Respiratory Syndrome Coronavirus 2 pandemic has challenged medical systems to the brink of collapse around the globe. In this paper, logistic regression and three other artificial intelligence models (XGBoost, Artificial Neural Network and Random Forest) are described and used to predict mortality risk of individual patients. The database is based on census data for the designated area and co-morbidities obtained using data from the Ontario Health Data Platform. The dataset consisted of more than 280,000 COVID-19 cases in Ontario for a wide-range of age groups; 0–9, 10–19, 20–29, 30–39, 40–49, 50–59, 60–69, 70–79, 80–89, and 90+. Findings resulting from using logistic regression, XGBoost, Artificial Neural Network and Random Forest, all demonstrate excellent discrimination (area under the curve for all models exceeded 0.948 with the best performance being 0.956 for an XGBoost model). Based on SHapley Additive exPlanations values, the importance of 24 variables are identified, and the findings indicated the highest importance variables are, in order of importance, age, date of test, sex, and presence/absence of chronic dementia. The findings from this study allow the identification of out-patients who are likely to deteriorate into severe cases, allowing medical professionals to make decisions on timely treatments. Furthermore, the methodology and results may be extended to other public health regions.

## Introduction

Since the outbreak of severe acute respiratory syndrome coronavirus 2 (SARS-CoV-2), also known as COVID-19, in Wuhan, China in December 2019, the virus has caused chaos, extreme and widespread illness and mortalities, and shutdowns of country-wide economies around the globe. As a measure of the sheer magnitude of the impacts, more than 11.9% of the US population have tested positive for the virus, as of January 25, 2021 (1). In Ontario, COVID-19 has infected 282,511 people and resulted in 6,614 deaths as of February 13, 2021 (2).

In response to the day-to-day challenges of managing the COVID-19 pandemic, massive efforts continue to be made to protect the integrity of hospital systems to allow both treatment of COVID-19 cases as needed, as well as to avoid having to delay elective surgeries. However, the growth in caseloads continues to be an ongoing major concern, needing improved predictions to use for capacity planning. To better understand and assess the capacity of health care systems to respond to the medical needs arising from COVID-19, enormous efforts are being expended involving the use of epidemiological and machine learning modeling, to understand the projection of caseloads of the virus. An example is an artificial neural network (ANN) model developed by Abdulaal et al. to predict a patient-specific, point-of-admission mortality risk to inform clinical management decisions (3). Their modeling analyzed patient features including demographics, co-morbidities, smoking history, and presenting symptoms of 398 patients and achieved an area under the curve (AUC) of 86% (3). However, while epidemiological modeling has significant value for certain questions, the parameters of these models are typically resolved through some estimation procedure and future case counts through different classes are identified. As an example, the SEIR model approach assigned individuals to the susceptible (S), exposed (E), infected (I), and recovered (R) classes (1, 2, 4). However, the potential to predict how positive tests for COVID-19 at health centers will translate to ICU cases and mortality are limited. This information will be critical as health systems in North America are rapidly approaching capacity.

With issues of the second wave of the pandemic ongoing at the time of submission of this paper, interest continues to grow in obtaining more comprehensive caseload and patient data and predictions. Given there are now more accurate data available from the first and second waves, inclusion of these data has considerable merit. Until now, data security and privacy issues have to-date limited the accessibility of alternate and detailed data sources. Through obtaining detailed COVID-19 pandemic-related data, more powerful artificial intelligence (AI) techniques can be feasibly applied, offering the ability to uncover and understand the value of alternative interpretation procedures.

This paper describes results arising from use of AI models based on data from Ontario Health Data Platform (22 February 2020–20 October 2020) with the aim to improve prediction of caseloads and mortality risks in patients throughout the province of Ontario, with a population of more than 14 million. The dataset includes extensive information from the first wave and the beginning of the second wave of COVID-19 cases including Canadian census information by designated area. The census location information is based on a size of approximately three blocks and hence is able to capture representation of ethnicity, income level, and other social differences, and can therefore be considered robust. The results from this paper may be extended further to other public health regions throughout the world. The paper is organized as follows: related works are discussed in section Related Works. Section Materials and Methods describes the materials and methods. Results and discussion are found in section Results and Discussion and then the paper is concluded in section Conclusions.

## Related Works

AI models can be employed in the prediction of patient-specific, point-of-admission mortality risks helping to inform clinical management decisions. Thus, using AI models, healthcare practitioners have ways to accurately predict the potential for an individual ending up in the Intensive Care Unit (ICU). As well, due to the very high AUC, AI models have the capability to consider a broad range of patient data, including demographics, co-morbidities, and much more, to predict patient-specific mortality risk following their hospital admission.

The emergence of “big data” in the early 2000s has proven very beneficial for public health investigations. The capabilities of AI modeling have been able to accurately predict numerous public health trends such as in-hospital mortality and detection of emerging epidemics (4–6). With the emergence of the COVID-19 global epidemic, big data and machine learning models have enormous potential to understand who, and where, people are most at risk.

Since March 2020, COVID-19 has spread around the globe, affecting every country and causing more than 2.1 million deaths worldwide (7). As a result, researchers have focused on COVID-19, to understand individuals most at risk as well as how to best distribute scarce resources. Identification of co-morbidities and creation of models to predict COVID-19 mortality using big data has significant potential.

Predictive analytics for assessing the in-hospital risk of patients and patient mortality have traditionally been limited to clinical decision rules (rules to reduce uncertainties in medical decision-making), but these approaches are not easily updated with new developments or data. Additionally, clinical decision rules may take years to identify and test and have been criticized for their lack of generally applicability to entire populations (4, 5). Hence, in the current situation, with the rapid spread of COVID-19, clinical decision support systems are still being developed for this particular virus (8).

Both Hernesneimi et al. and Taylor et al. note that one of the biggest challenges in developing models to predict mortality is assembling quality data (4, 5). However, this is outweighed by the benefits of a machine learning model, which provides improved performance and ability to be generalized to entire populations, or readily adapted to new populations (9). As well, AI models are updateable over time to recognize changing trends in medical care, all of which are attainable dimensions, if provided the large datasets (4, 5).

Rodriguez-Nava et al. used a random forest algorithm that predicted ICU admissions with an AUC of 0.82 and mortality with an AUC of 0.70 (10). Similarly, Jimenez-Solemm et al. used a random forest machine learning model using a Danish dataset with 3,944 COVID-19 patients that predicted ICU admissions with an AUC of 0.820, mortality with an AUC of 0.902, hospital admission with an AUC of 0.820 and ventilator treatment with an AUC of 0.815 (11). They found age and BMI the most important features for predicting hospital admission and ventilator treatment (11). Among all models, the parameters that were determined to have the highest severity of COVID-19 were heightened if male, had hypertension, and presence of dementia (11).

A study by Schöning et al. aiming to distinguish between severe and non-severe COVID-19 also found hypertension, along with diabetes mellitus (Type 2) and renal impairment to be prognostic of severe disease (12). Schöning et al. used a variety of machine learning models which were trained using data from the first wave in Switzerland and externally validated using data from the second wave (confirming findings to be generalizable) and achieved an accuracy of AUC values ranging from 0.86 (decision tree induction) to 0.96 (support vector machine) (12).

Kim et al. used Korean National Health Insurance data to identify co-morbidities and factors that increase mortality using multivariate logistic regression analysis with a confidence interval of 95% (13). A total of 9,148 COVID-19 patients, including 130 fatalities, were linked to their national health insurance record data, considering 298 medical conditions as co-morbidities (13). Williamson et al. performed a similar analysis on behalf of NHS England, with a sample size of 10,926 COVID-19 related deaths (14). Using a multivariable Cox model, Williamson et al. were able to estimate hazard ratios for characteristics such as smoking status, obesity, sex, age, deprivation, heart disease, etc. with a 95% confidence interval (14). Both studies identified age, deprivation, diabetes, bronchitis and severe asthma as top risk factors for COVID-19 (12, 13). Additionally, Kim et al. found that dental disorders were associated with high co-morbidity risk (13).

Machine learning has been found to predict mortality more accurately than non-machine learning models (4, 5). For example, mortality due to acute coronary syndrome is reliably predicted using the GRACE score, based on patients' variables in admission to the hospital. A machine learning model developed by Hernesneimi et al. achieved greatly improved results compared to traditional GRACE score (4). The machine learning model was developed using XGBoost and included phenotype data about the patient as well as hospital admission variables (4). The machine learning model outdid the traditional GRACE score method with an AUC of 0.904 compared to an AUC of 0.802 for GRACE score (4). Another example is a machine learning model developed by Taylor et al. to predict in-hospital mortality of patients with sepsis (5). This model considered 500 clinical variables including demographic information such as age, sex, insurance and employment status as well as health status and medical test results. Random Forest was reported to be a relatively interpretable option and produced the best results when compared with XGBoost, logistic regression or CART models (5).

A similar study from Zheng et al. used machine learning models to evaluate patients and predict rapid deterioration of in-hospital COVID-19 patients to determine triage priority (15). The model developed by Zheng et al. used the results of blood tests from 601 COVID-19 patients to predict deterioration up to 12 days before death occurred and reported that decreased lymphocyte count and increased lactate dehydrogenase levels, c-reactive protein levels and neutrophil counts, all corresponded with an increased likelihood for rapid deterioration (15). The best results were found using XGBoost and achieved an AUC of 0.953 for a small dataset (15). In addition, the illness trajectory (moderate, severe, critical – states as defined by Israeli Ministry of Health) of COVID-19 patients was predicted by Roimi et al. with an AUC of 0.88 using only patients' age, sex and day-by-day clinical state using a multistate Cox regression-based model (16).

Estiri et al. used nested generalized linear models to predict mortality using electronic health records (EHRs) of 16,709 COVID-19 age-separated cohorts from Mass General Brigham (a Boston-based non-profit hospital) (17). The data included were from the beginning of the EHR up to 14 days prior to the positive polymerase chain reaction (PCR) test to ensure COVID-related medications were not included in the model as risk factors (17). The model which included patients under the age of 45 had an AUC of 0.898, 0.789 in the 45–65 cohort and 0.753 in the 65–85 cohort, thus able to provide relatively high accuracy by relying only on data already stored in EHRs (17).

A prognostic machine learning model created by researchers from the Department of Decision Medicine at the University of Maastricht in The Netherlands reported the severity of COVID-19 in a patient (8). The model is based on the data from 299 patients' blood-test data and age and is readily available as an application (8). A similar application developed by ClosedLoop.ai determines a “vulnerability score” for an individual's susceptibility to severe COVID-19 illness (18).

Machine learning models have also been trained to predict rapid deterioration due to COVID-19 and even detect COVID-19 from lung X-Rays (19, 20). Zhu et al. used deep transfer learning to determine COVID-19 severity from X-rays and Elgendi et al. used deep neural networks to differentiate COVID-19 patient X-rays from other types of pneumonias (19, 20). Both methods used convolutional neural networks to a high degree of accuracy, reducing the need for radiologists (who may be in short supply in low-resource clinics) to read x-rays (19).

Mehta et al. used caseload and mortality data from early in the COVID-19 pandemic (March 2020), to determine the vulnerability of a county in the US to a large COVID-19 outbreak with high mortality (21). Mehta et al. used XGBoost to train a model that would predict which counties in the US were most at-risk using county-level population statistics such as age, gender, and density as well as CDC data for the health within a county (21). They reported that population size of a county was by far the most important variable, with population density, longitude, hypertension prevalence, chronic respiratory mortality rate, cancer crude rate, and diabetes prevalence, also playing large roles (21). While Mehta et al. do not state which counties are found to be the most at-risk, COVID-19 has reached virtually all parts of the U.S., including both rural and urban areas (21, 22).

As widely apparent from the numerous information venues, the magnitude of the impacts to people and the economies around the world are staggering. Hence, there is enormous interest in using available data, and in the development of models, that can combine available information in any manner to improve predictions of caseloads, supporting alternative strategies for predicting patient-specific mortality risk.

## Materials and Methods

### Dataset Description

For this research, to ensure adherence to strict security protocols, extensive data for 57,390 individual cases from Ontario Health Data Platform where data related to positive COVID-19 tests were collected between 22 February and 20 October 2020 were obtained for use in machine learning modeling. The dataset contained epidemiological and demographic information, recovery/mortality outcome information and co-morbidities of individuals residing in Ontario at census level. The attributes which proved most useful in the machine learning and statistical models are indicated in Table 1. Co-morbidities and age were collected from patient health records as of January 1, 2020; hence diagnoses of other medical conditions after this date were excluded. Of the 57,390 cases included in the dataset, 2,822 patients died of COVID-19 and the remaining 54,568 either recovered from COVID-19 or are still hospitalized.

**Table 1:** Characteristics of 57,390 Ontario residents with COVID-19.

**Variable**	**Description**	**Range of values**
Age	Age in years, as of Jan 1, 2020	0–105
Test date	Test date	Feb 22–Oct 20
Sex	Indicator Variable for sex	26,861 (M = 1, F = 0)
Hypertension	Chronic hypertension, as of Jan 1, 2020	15,778 (0, 1)
LTC resident	LTC resident, as of Jan 1, 2020	5,179 (0, 1)
Chronic_dementia	Chronic dementia diagnosed, as of Jan 1, 2020	4,746 (0, 1)
Chronic_odd	Chronic diabetes diagnosed as of Jan 1, 2020	9,002 (0, 1)
Ethnic concentration quint.	Calculated from ontario marginalization index, based on census designation. Refers to Visible minorities and/or recent immigrants (0–5 ranging from least diverse to most diverse)	(0–5)
Commuter concentration quint	% of people that commute within Census designated area - converted to quintiles (5 being the highest, 0 referring to missing DA info).	(0–5)
Median income quint.	Median income within census-designated area - converted to quintiles (0–5 ranging from Lowest income to highest income, 0 referring to missing DA info).	(0–5)
Charl	Charlson co-morbidity index. Only 2,059 patients with charl above 0.	(0–10)
Household size quint.	Avg. household size within Census-designated area - converted to quintiles (5 being the Highest, 0 = missing DA info).	(0–5)
CKD	Chronic kidney disease.	2,523 (0, 1)
Cancer	Cancer index	2,995 (0–1)
Chronic_copd	Chronic obstructive pulmonary disease	4,030 (0–1)
Chronic_asthma	Asthma	9,100 (0–1)
Chronic_chf	Congestive heart failure	2,257 (0–1)
Stroke	If patient suffered a stroke previous to Jan 1, 2020	1,016 (0–1)
Cardiac ISCH	Cardiac ischemia	1,916 (0–1)
Rural	Indicator if a patient lives in a rural residence	1,746 (0–1)
Chronic_ra	Rheumatoid arthritis	567 (0–1)
Tia	Transient Ischemic Attack	722 (0–1)
Immuno_comp	Immuno-compromised	237 (0–1)
Thala	History of Thalassemia	36 (0–1)
Cases recovered		54,568
Cases died		2,822

Several input variables were derived using 2016 census data for the designated area of the individual patients. Census data in Canada are collected at postal code level and hence, represent approximately at three city block intervals. Census data relied upon included: ethnic concentration (of residential area), commuter concentration, median income and household size (these values are unlikely to change significantly between date of census and start of pandemic). These values were converted into quintiles (division of the population into 5 equal groups according to the distribution of input variables) with “1” being the lowest quintile, and “5” being the highest. Long-term care residents (LTC) did not include census-designated area information and therefore were represented with a zero value in the AI modeling. As well, for case-specific data for which substantial data were not available, those cases were removed from the modeling prior to undertaking the modeling (decreasing the 280,000 data to 57,390 cases actually used in the modeling) since the remaining cases represented a very substantial dataset for analyses. Individuals with missing data were not included in the analyses, however, for LTC residents' variables derived based on postal code, such as income, ethnic concentration, household size, and commuter percentage, were coded as zero since postal code information was not available for these individuals.

### Model Development

This study compared three machine learning models and one statistical model. The following models were employed: artificial neural network (ANN), Random Forest (RF), extreme gradient boosting decision tree (XGBoost) all of which are tree structure machine learning models, and logistic regression (23–25). These models were adopted due to their high accuracy in binary classification problems as well as their prevalence/adoption in previous literature. The hardware used for developing the models included: a virtual server from OHDP specifically to run the code, 8 virtual CPUs and 128 GB of RAM, all running on Centos 7 Operating System. The software used included: R for coding and Rstudio as the integrated development environment (IDE).

### Data Processing

Prior to model calibration, the dataset was randomly split into two segments, namely an 80% training dataset as an operational characterization and a 20% testing dataset where each model was calibrated using the training dataset and assessed for accuracy using the testing dataset. A grid search approach was used to adjust the hyperparameters of the models using a 10-fold cross-validation technique, repeated three times per model and optimized to produce the maximum area under the receiver operating characteristic curve (Area Under Curve, or AUC).

The logistic regression's input variables were chosen by a stepwise Akaike Information Criterion (AIC) function (23). The computer programming language R was used to develop and analyze all models (26). The final predicted outcome, recovered or died, was determined by the probability of mortality for each recorded case outcome generated by the models, based on a 50% threshold value ensuring highest total accuracy.

## Results and Discussion

As a measure of accuracy, the Receiver Operating Characteristic curve (ROC) was calculated for each model. The Area Under the ROC curve was used to provide the basis of comparison between each model. Since an AUC value approaching “1” indicates high model accuracy while a value of 0.5 represents a model that is no better than random change, Table 2 demonstrates that all models utilized show strong predictive values.

**Table 2:** Comparison of models employed in the base case analyses.

**Model**	**AUC**
Logit	0.9518
**XGboost**	**0.956**
Random forest	0.948
Neural net	0.9475

*The bold values represent the accuracy for the model (i.e., XGBoost) which is used primarily in this paper to explore the importance of variables*.

All models developed have very high AUC values (>0.94). The most accurate model is XGBoost which has an AUC of 0.956. To the best of the authors' knowledge, this is the highest accuracy reported for COVID-19 mortality prediction models published to-date using Canadian data.

Although the AUC is a useful metric in understanding the overall accuracy of a binary prediction model, that metric does not provide specific accuracy for each class (accuracy in predicting survival vs. accuracy in predicting mortality). To highlight the accuracy of both predictions, a confusion matrix was developed. The confusion matrix as developed and highlighted in Table 3, compares the prediction accuracy of the XGBoost model with the reference (or actual outcome) of COVID-19 patients within the test dataset, assuming any patient with a predicted risk score below 0.5 will survive.

**Table 3:** Confusion matrix and statistics.

**Prediction**	**Alive**	**Dead**
Alive	10,710	353
Dead	203	211

The confusion matrix indicates the model is extremely accurate in predicting which patients will survive, with 97% of the alive predictions actually surviving. The mortality prediction accuracy is substantially lower, with 49% of predicted deaths actually occurring. Even at 49%, the mortality prediction still provides a strong indicator on whether a COVID-19 patient will die and is helpful in strategic planning of possible medical caseloads, in terms of potential overload of the medical system.

It is noted that the mortality prediction accuracy of the modeling could be improved by increasing the risk score threshold, but this would also increase false negative predictions. Various methods have been created in an attempt to quantify the variable impact but many of them are flawed when using tree structure machine learning models (which includes XGBoost, Random Forests, decision trees, etc.). While the values could be adjusted to make a new set point, the models are quite accurate and are best represented by the AUC, meaning it is a good indicator of the actual risk of mortality. Overall, the results indicate the risk score predicted by the XGBoost model provides very strong insights regarding the outcome for Ontario COVID-19 patients, including the ability to plan for possible medical caseloads.

### Variable Importance

Since the XGBoost model is the most accurate mortality prediction model developed for COVID-19 patients in Ontario, there is strong merit in investigating which variables are the most important and quantify how these variables contribute to the final predictions. To explore the impact of each variable on the final prediction, SHapley Additive exPlanations (SHAP) values have been used.

SHAP values determine the importance of a feature by comparing a model prediction with, and without, the feature for each observation within the training data. The SHAP values are calculated using SHAPforXGBoost R package and present the variable contribution on a log-odds scale (logarithm of the ratio of high mortality risk to low mortality risk) (27).

**Figure 1:**
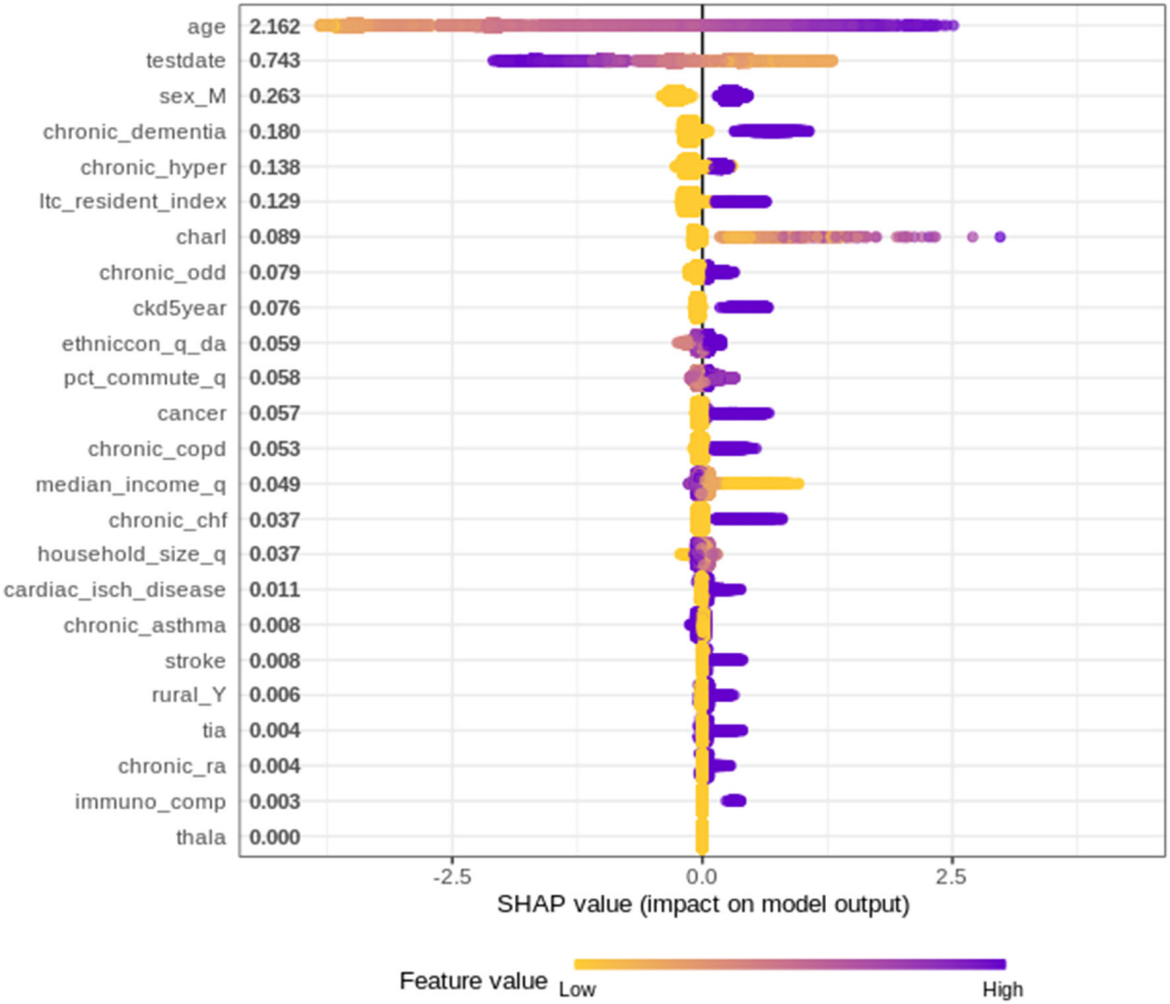
SHAP summary plot for XGBoost model.

Figure 1 plots the SHAP value for each individual patient within the training dataset by input variable. The input variables, as listed on the y-axis, are ranked from most important (at the top) to least important (at the bottom) with their mean absolute SHAP value indicated next to the name. The X axis represents the SHAP value associated with each variable and patient within the training dataset (i.e., there is a plotted point for each case based on the influence that variable has on the prediction of that case). The color indicates whether the individual patients' input variable value was high (purple) or low (yellow). For example, in Figure 1 a “high” age has a high and “positive” impact on predicting mortality. The “high” comes from the purple color and the “positive” impact is shown in the X axis. Note, a range of SHAP values can exist per input variable value based on the SHAP values calculated for each observation and how they independently contribute to the machine learning model's predictions.

Overall, 24 variables were identified/ranked, with age as being unquestionably the most important variable for the XGBoost model. As a patient's age increases (approaches purple) the SHAP value impact increases, with a very high age being associated with an additional 2.5 increase in log-odds. The date of when someone tested positive for COVID-19 also demonstrated a strong impact on overall mortality risk, indicating as the positive test date increases (i.e., later in the pandemic) the risk of mortality decreases. The importance of the date when someone tested positive is very likely due to improved understanding of treatment options by the medical systems. Since the start of the pandemic in March 2020, health care workers and researchers worked fast to try to find the most effective ways to treat and prevent COVID-19 (e.g., including the use of antivirals and immune modulators, such as remdesivir, hydroxychloroquine, and dexamethasone) (28, 29). This finding described herein is supported by the European Medicines Agency indicated in June 2020 that it was discussing 132 potential treatments with developers, showing the rapid speed with which drugs and therapies against the virus are being developed (29, 30).

Other variables of high importance in terms of predicting the outcomes included “sex,” and chronic dementia, etc. on through the list of 24 variables, as depicted in Figure 1.

## Conclusions

The impact of COVID-19 has been felt throughout populations around the world. In Ontario, COVID-19 has infected over 280,000 people with over 6,000 deaths (2). As the people of Ontario experience the second wave of the pandemic, the number of deaths associated with the pandemic will continue to increase. Understanding which Ontarians are most at risk will be important in determining how the medical system can most effectively deal with the pandemic, implement effective intervention strategies, and refine vaccination priorities.

This paper described a model with an accurate mortality prediction that can be used to assist Ontario's medical system in combating this pandemic and further highlight the risks faced by individual patients. By including patient demographics, co-morbidities, geographic, and census-based attributes, the XGBoost model developed surpassed all other Ontario mortality predictions published to date, with an AUC of 0.956; all four modeling approaches provided high AUCs where findings resulting from using logistic regression, XGBoost, ANN and RF, all demonstrate excellent discrimination (area under the curve for all four modeling approaches exceeded 0.948).

Findings related to the importance of 24 variables in characterizing mortality based on SHAP values, the importance of the variables found the most important parameters in order of importance were age, date of test, sex and chronic dementia, providing guidance to the medical professionals to identify the highest risks for out-patients who are likely to deteriorate into severe cases with the features indicating the risk of mortality.

## Data Availability Statement

The data analyzed in this study is subject to the following licenses/restrictions: the dataset obtained from the Ontario Health Data Platform limits access to these data sets to trustworthy researchers, who are bound by law, ethics, the terms of their employment, and their research grants to protect the privacy and confidentiality of the data they work with. Requests to access these datasets should be directed to https://ohdp.ca/pre-application-form/.

## Author Contributions

EAM, BS, JY, and SAG: conceptualization. BS: methodology and software. BS, EAM, JY, SAG, and BP: validation. BS, EAM, JY, SAG, and BP: formal analysis. EM: investigation. BS and EAM: data curation. BS, EAM, and BP: writing—original draft preparation. BS, EAM, JY, SAG, and BP: writing—review and editing. EAM: supervision, project administration, and funding acquisition. All authors have read and agreed to the published version of the manuscript.

## Conflict of Interest

JY is employed by the company Adastra Corporation however Adastra has no financial or other conflicts of interest in this work. The remaining authors declare that the research was conducted in the absence of any commercial or financial relationships that could be construed as a potential conflict of interest.
