# Corrigendum: Identification of Variable Importance for Predictions of Mortality From COVID-19 Using AI Models for Ontario, Canada

**DOI:** 10.3389/fpubh.2021.759014

**Published:** 2021-09-23

**Authors:** Brett Snider, Edward A. McBean, John Yawney, S. Andrew Gadsden, Bhumi Patel

**Affiliations:** ^1^School of Engineering, University of Guelph, Guelph, ON, Canada; ^2^Adastra Corporation, Toronto, ON, Canada

**Keywords:** artificial intelligence, XGBoost, SHapley, COVID-19, mortality

In the original article, there was an error in the **Acknowledgments**, the dataset we analyzed requires very specific language regarding **Acknowledgments**.

The original statement read: “The authors gratefully acknowledge support from the Ontario Ministry of Health, Canada who supplied critical medical data for this study as well as the assistance of Ontario Health Data Platform for handling the security protocols pertinent to protecting the privacy of the data.”

However, it should read:

“This study was supported by ICES, which is funded by an annual grant from the Ontario Ministry of Health (MOH) and the Ministry of Long-Term Care (MLTC). This study was supported by the Ontario Health Data Platform (OHDP), a Province of Ontario initiative to support Ontario's ongoing response to COVID-19 and its related impacts. The analyses, opinions, results, and conclusions reported in this paper are those of the authors and are independent from the funding sources. No endorsement by ICES, the OHDP, its partners, or the Province of Ontario is intended or should be inferred. Parts of this material are based on data and/or information compiled and provided by CIHI. However, the analyses, conclusions, opinions, and statements expressed in the material are those of the author(s), and not necessarily those of CIHI.

These datasets were linked using unique encoded identifiers and analyzed at ICES.

REB: The use of the data in this project is authorized under section 45 of Ontario's Personal Health Information Protection Act (PHIPA) and does not require review by a Research Ethics Board.

Access to datasets: The dataset from this study is held securely in coded form at ICES. While legal data sharing agreements between ICES and data providers (e.g., healthcare organizations and government) prohibit ICES from making the dataset publicly available, access may be granted to those who meet pre-specified criteria for confidential access, available at www.ices.on.ca/DAS (email: das@ices.on.ca). The full dataset creation plan and underlying analytic code are available from the authors upon request, understanding that the computer programs may rely upon coding templates or macros that are unique to ICES and are therefore either inaccessible or may require modification.

OCR: Parts of this material are based on data and information provided by Cancer Care Ontario (CCO). The opinions, results, view, and conclusions reported in this paper are those of the authors and do not necessarily reflect those of CCO. No endorsement by CCO is intended or should be inferred.

ODB: We thank IQVIA Solutions Canada Inc. for use of their Drug Information Database.

Ontario Community Health Profiles Partnership (OCHPP): OCHPP created Ontario Marginalization Index (ON-Marg) which is a source for this paper as ON-Marg is used to understand inequalities in health and other social problems related to health among either population groups or geographic areas across Ontario.

Statistics Canada: Postal Code Conversion File and census data were adapted from Statistics Canada. This does not constitute an endorsement by Statistics Canada of this product.”

The authors apologize for this error and state that this does not change the scientific conclusions of the article in any way. The original article has been updated.

## Publisher's Note

All claims expressed in this article are solely those of the authors and do not necessarily represent those of their affiliated organizations, or those of the publisher, the editors and the reviewers. Any product that may be evaluated in this article, or claim that may be made by its manufacturer, is not guaranteed or endorsed by the publisher.

